# A Case of Atypical Acute Limb Ischemia and Concurrent Fournier's Gangrene

**DOI:** 10.7759/cureus.81224

**Published:** 2025-03-26

**Authors:** Rooshan Arshad, Jenny Bui, Loay S Kabbani, Sina Khoshbin

**Affiliations:** 1 Department of General Surgery, Wayne State University School of Medicine, Detroit, USA; 2 Department of General Surgery, Henry Ford Health System, Detroit, USA; 3 Department of Vascular Surgery, Henry Ford Health System, Detroit, USA; 4 Department of Acute Care Surgery, Henry Ford Health System, Detroit, USA

**Keywords:** acute care surgery and trauma, acute limb ischemia, acute trauma care, fournier’s gangrene, multidisciplinary approach, necrotizing soft tissue infection

## Abstract

Fournier’s gangrene is a severe necrotizing soft tissue infection involving the perineal, perianal, and genital areas, where early and aggressive surgical debridement is the key determinant for survival. Acute limb ischemia (ALI) occurs when vascular compromise threatens a limb, often requiring revascularization or amputation. A synergistic pathophysiological effect between Fournier’s gangrene and ALI can be appreciated as infection promotes microthrombi, which leads to vessel occlusion, exacerbating the soft-tissue gangrene and local ischemia. Thus, fulminant Fournier’s gangrene promotes a hypercoagulable state, which may result in ALI. A 63-year-old African American male with hypertension, hyperlipidemia, and a 35-pack-year smoking history presented with left lower extremity pain, scrotal swelling, and absent pedal pulses. Color Doppler showed an occlusion proximal to the left popliteal artery, and CT confirmed Fournier’s gangrene extending to the presacral area. The patient was taken emergently to the operating room for an attempt at reperfusion of the left lower extremity. This was unsuccessful due to the extent of vascular disease. Extensive surgical debridement of the genitals and perineum was also performed. Subsequent trips to the operating room for further debridement were required. Ultimately, a left above-knee amputation was performed. Despite source control of his infection, the patient suffered an aspiration event on hospital day seven, which led to hypercarbic respiratory failure and cardiac arrest. This resulted in a poor neurologic outcome, from which he did not recover. The patient was transitioned to comfort measures and expired on hospital day 20. The rarity of concurrent Fournier’s gangrene and ALI presents a unique clinical question, as one must decide which problem should be treated first on the operating table. This complex management decision requires a multidisciplinary approach accounting for the clinical severity of both disease processes.

## Introduction

Patients with acute limb ischemia (ALI) present with six cardinal characteristics: pain, paresthesia, pallor, pulselessness, poikilothermia, and paralysis. Paralysis portends the worst prognosis, often requiring limb amputation [1]. Surgical thromboembolectomy, collateral/bypass grafting, endovascular revascularization, thrombolytic therapy, and percutaneous transluminal angioplasty (PTA) are the major treatment options for ALI [2,3]. ALI can stem from various etiologies, thrombotic, embolic, or traumatic, and is reported to have a higher rate of occurrence in patients with comorbid conditions such as hypertension, atherosclerotic disease, diabetes, and congestive heart failure. ALI patients tend to have low medical optimization due to the acuity and, therefore, a high complication rate. A five-year mortality rate is estimated to be 36-65%, dependent on patient comorbidities and the revascularization technique employed [4].

Fournier’s gangrene is the necrotization of skin and soft tissue infection of the perineal, perianal, and genital areas with an incidence of 1.6/100,000 males annually [5,6]. If left untreated, Fournier’s gangrene can progress rapidly to muscular involvement, spreading to the scrotum, penis, and along the muscle of the abdominal wall and thighs. It comes with a high mortality rate of 20-40% and is managed with emergent surgical debridement, with time to surgery being a determinant factor for mortality and broad-spectrum antibiotic therapy [7,8].

ALI and Fournier’s gangrene share similar risk factor profiles such as advanced age, hypertension, heart disease, diabetes, vascular disease, kidney disease, and smoking history [2,9]. These comorbidities increase the incidence of morbidity and mortality in ALI and Fournier’s gangrene. Furthermore, extensive gangrene on top of ALI creates an ouroboros-like dilemma. Gangrene and infection promote microthrombi, which promote vessel occlusion, which exacerbates the soft-tissue gangrene and infection, and so forth [10]. Perfusion insufficiency and the gangrenous infection then simultaneously threaten limb and life.

We present a case of clinically extensive Fournier’s gangrene precipitating with acute limb ischemia. This case is important for clinicians because the concurrent presentation of these conditions is rarely reported in the literature. Consequently, this represents a significant diagnostic and therapeutic challenge.

## Case presentation

A 63-year-old African American male with a history of hypertension, hyperlipidemia, and a 35-pack-year smoking history presented to a free-standing emergency room with a one-day history of a cool left lower extremity, numbness, and pain. He also noted tingling and scrotal discomfort. This patient had no known history of any vascular disease and denied any prior symptoms of claudication or limb pain. 

**Figure 1:**
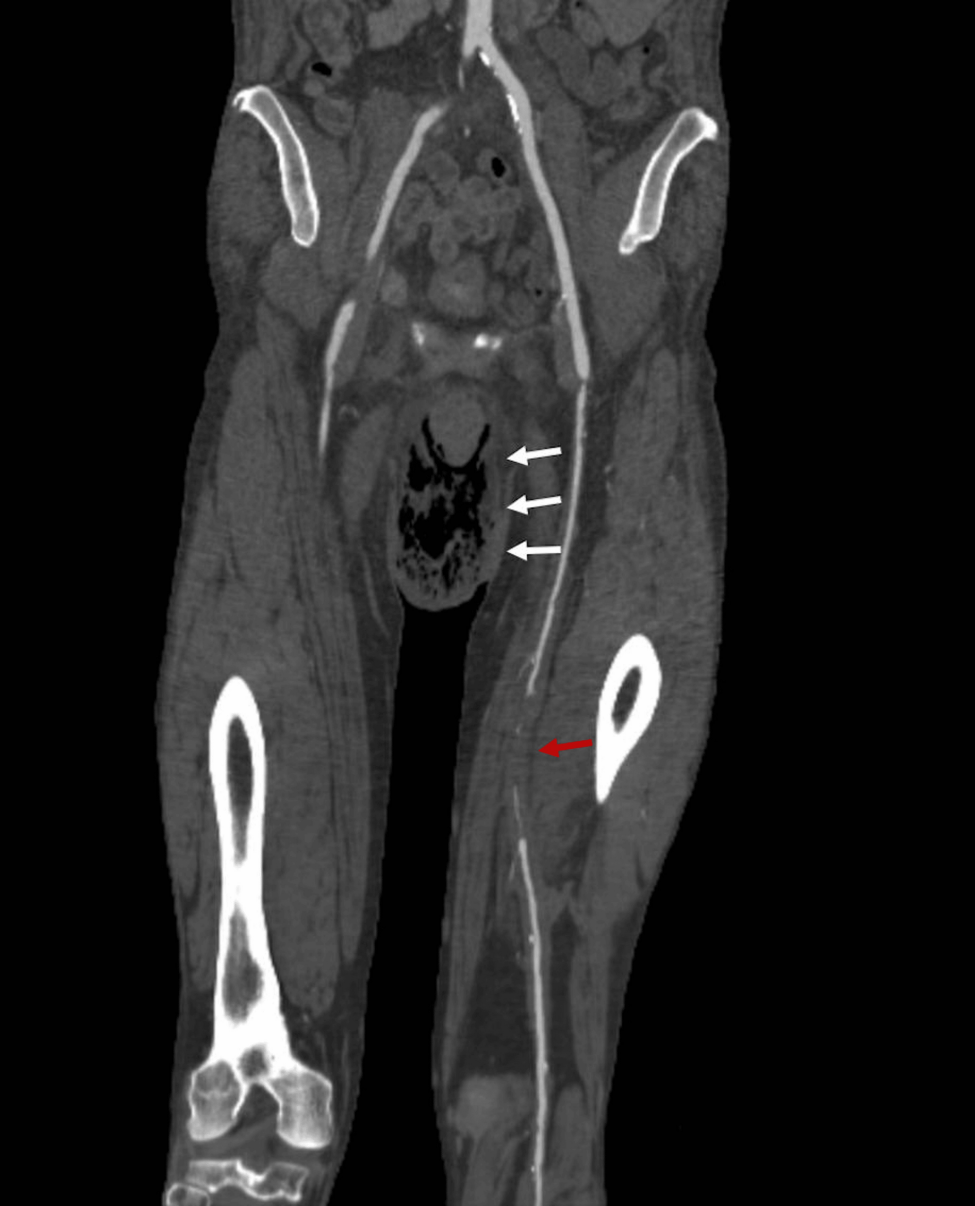
CT findings. CT schematic showing the extent of vascular disease in the left leg and gas in the perineal and scrotal soft tissue (white arrows). Occlusion at the distal left superficial femoral artery (red arrow).

On arrival to the emergency room, initial vitals were unremarkable. Blood pressure of 136/48, heart rate of 97, temperature of 97.3°F, respiration rate of 16. The patient was reported to be in supraventricular tachycardia, which was self-limited and resolved within 10 minutes of presentation. He reported diminished sensation and an inability to ambulate with his left foot for one day. On exam, the patient had non-palpable left dorsal pedis and popliteal pulses with palpable bilateral femoral pulses. Pubic crepitus and an enlarged, swollen scrotum with purulent drainage were also noted. Pertinent labs are shown in Table 1. Color Doppler at the time showed occlusion proximal to the left popliteal artery. The Rutherford classification was assessed to Class IIb, given the sensory loss involving more than the toes, and he was able to move his left lower extremity with moderate motor weakness, and the inaudible arterial Doppler signals [11]. CT-abdomen-pelvis showed extensive gas extending from the posterior inferior buttock to the ilioischial fossa on the left, within the bilateral scrotum (Figure 1). Gas was also found in the anterior inferior abdominal wall and presacral retroperitoneum.

**Table 1:** Pertinent lab values obtained during the initial ER visit. WBC: white blood cells; ANC: absolute neutrophil count; PT: prothrombin time; INR: international normalized ratio

Lab	Results	Reference range
WBC	15.6 K/uL	3.8-10.6 K/uL
ANC	14.9 K/uL	2.5-7 K/uL
Lactate	4.3 mmol/L	<2.1 mmol/L
PT	18 sec	11.5-14.5 sec
INR	1.5	0.8 -1.1

**Figure 2:**
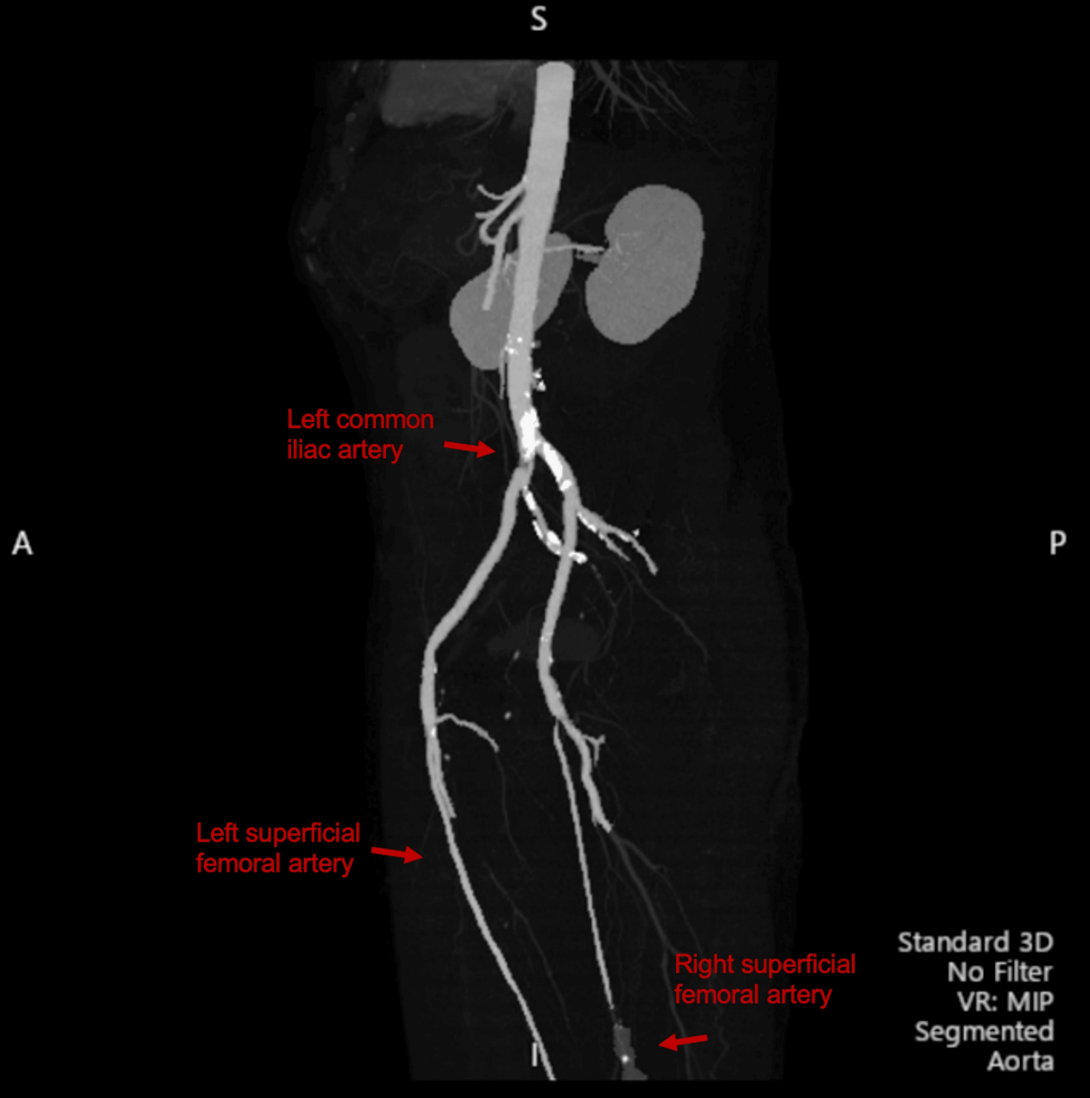
CT-angiography findings. The lower extremities revealed a large acute on chronic thrombus in the distal left common iliac artery. A long segment of complete occlusion of the distal right superficial femoral artery with reconstitution via collaterals supplied by the profunda as it entered the adductor hiatus with distal perfusion.

**Figure 3:**
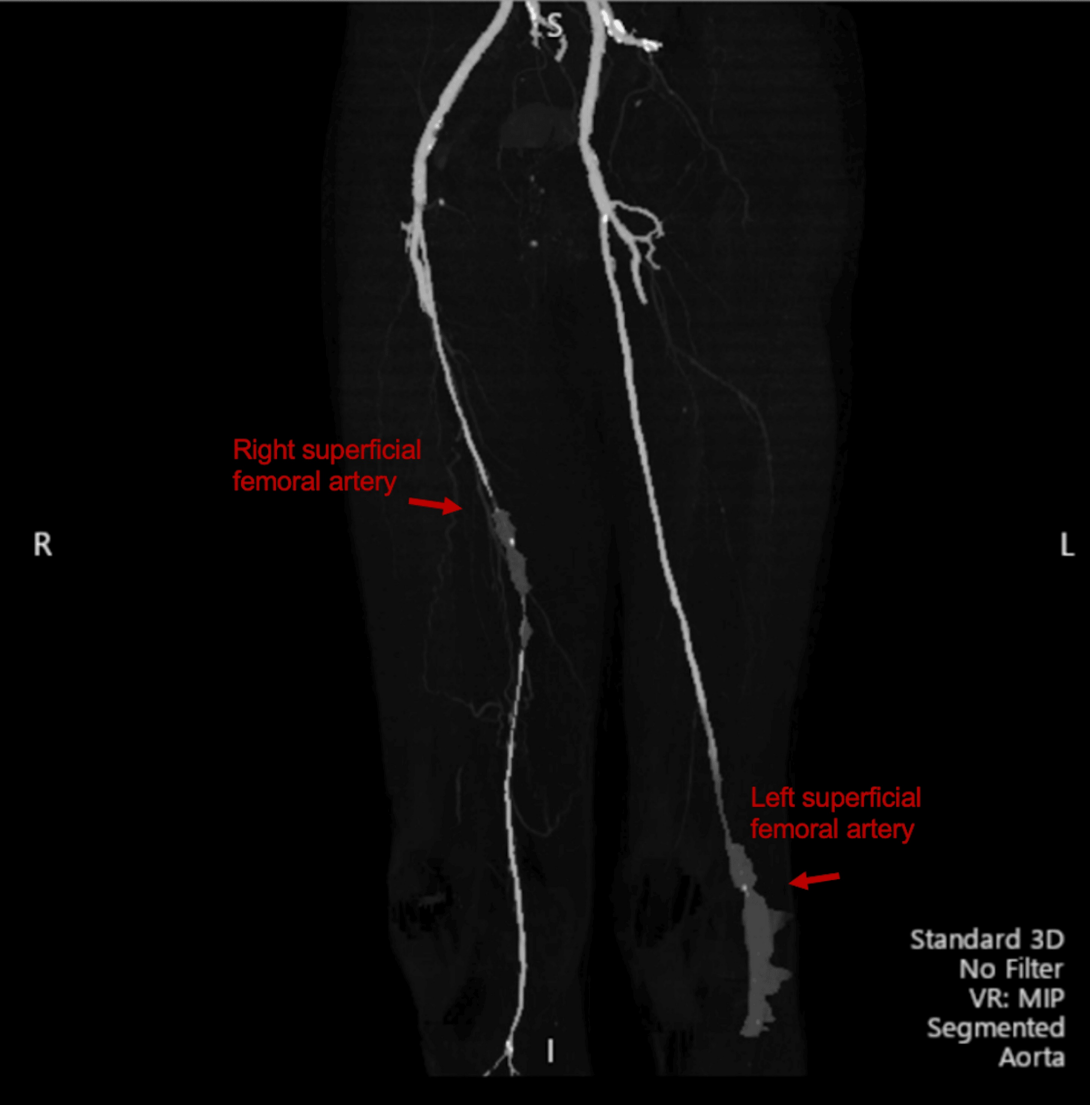
CT-angiography scan findings. Revealed greater occlusion of the right femoral profunda femoris artery in the right leg, compared to the thrombus occlusion of the left cold limb. Occlusion of the left profunda and popliteal artery without distal perfusion in the left leg is suspicious for a mixed calcified thromboembolic source.

Clinical presentation and imaging were diagnostic of Fournier’s gangrene. CT-angiography revealed extensive aortoiliac occlusion of the distal aorta and acute thrombus originating from a non-occlusive atheromatous plaque extending into the iliac arteries (Figure 2). An occlusion at the left profunda femoris artery and popliteal artery was also seen without distal perfusion (Figure 3). The patient was started on a heparin infusion and taken to the operating room with acute care and vascular surgery.

**Figure 4:**
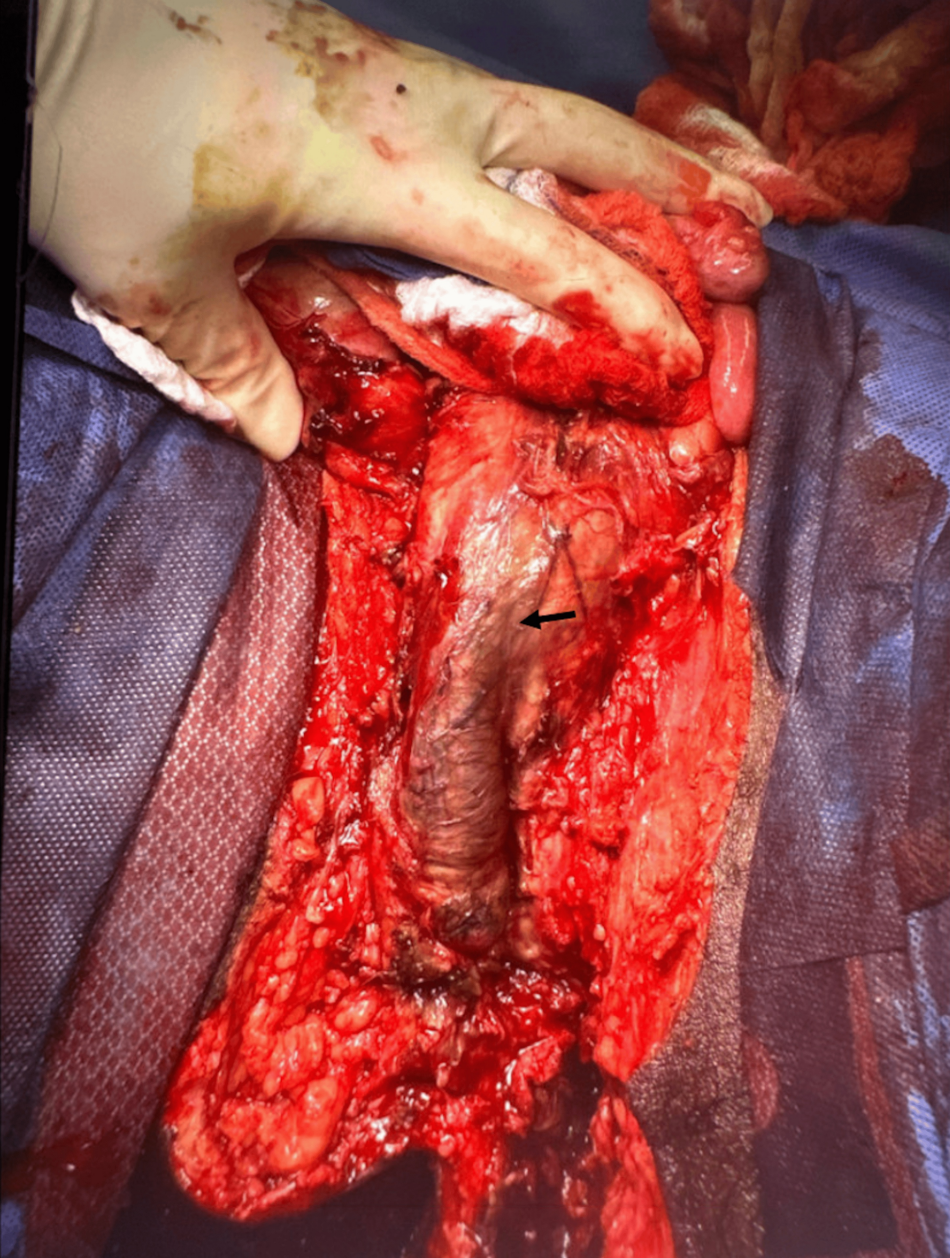
Perineal and scrotal defect after first debridement of necrotic tissue. Penile shaft: black arrow

The operation began with attempts at revascularization by left femoral artery cut-down, left popliteal artery cut-down, and thromboembolectomy, followed by a prophylactic fasciotomy. The left profunda femoris artery embolectomy was successful. However, both endovascular and open attempts at reperfusion of the left popliteal occlusion were unsuccessful due to complete occlusion of all runoff arteries and prolonged ischemia time. A four-compartment left leg fasciotomy was performed. Subsequently, the patient underwent penile shaft, left scrotum, and perineum debridement. Extensive liquefactive necrosis was noted (Figure 4). The patient underwent multiple debridements over the following days.

Once it was deemed that no further debridement was necessary, the patient underwent an above-knee amputation, and his exposed testes were placed back into a pocket of subcutaneous tissue within his groin. His hospital course was complicated by two episodes of stable supraventricular tachycardia (SVT), which were treated with one dose of adenosine each time. An echocardiogram showed no abnormalities. At this point, the patient was awake, recovering well, and improving each day.

On hospital day seven, the patient aspirated, resulting in hypercarbic respiratory failure. He suffered a cardiac arrest with pulseless electrical activity for 30 minutes. The patient was resuscitated but never recovered neurologically beyond a Glasgow Coma Scale (GCS) of 8T. Discussions with the family on the patient’s poor prognosis were held, and ultimately, he was transitioned to comfort care and expired.

## Discussion

Fournier’s gangrene (FG) can follow an insect bite, be triggered by a scratch, or poor hygiene practices, and sometimes occur without any clear inciting event [5,6]. If left untreated, Fournier’s gangrene can spread to involve the scrotum, penis, and the anterior abdominal wall. Testes are usually spared due to their intra-abdominal blood supply. However, orchiectomy for non‐viable testes is eventually required [12]. Patients who survive have an elongated recovery period, multiple reconstructive surgeries, and often suffer sexual and urological disabilities [9,12].

The etiology of ALI is typically embolic or in situ thrombosis. The more frequent of the two causes is embolic, but it can also follow traumatic vascular injury [13]. Not limited to FG, necrotizing fasciitis has been reported to contribute to the creation of septic emboli leading to multifocal necrotizing fasciitis [14]. The patient's multiple negative echocardiographs suggest a mural thrombus secondary to the transient arrhythmia or large septic emboli secondary to the developing sepsis or endocarditis, which were all unlikely. The imaging points to the patient being an undiagnosed vasculopath given the bilateral vessel partial occlusions. Thus, the likeliest mechanism of the acute on chronic thrombus on the left side, the side with lesser vessel occlusion compared to the right, was due to local septic emboli precipitation [14]. In our patient, we suspect the suppurative nature of the severe infection precipitated microthrombi and inflammation, leading to a hypercoagulable state and thrombus formation and propagation. The thrombus likely embolized, causing total occlusion of the infrapopliteal artery and severe ischemia, as well as embolization into the profunda femoris artery.

Due to the rarity of concurrent Fournier’s gangrene and ALI, the question of which problem should be treated first on the operating table is not one commonly asked. Once the site of occlusion is confirmed via CT-angiography and the extent of infection is established, determining which one should be treated first is situational and dependent on the severity of sepsis. One way to evaluate severity is via Fournier’s gangrene severity index (FGSI) [15]. FGSI at presentation for our patient was calculated to be 8; an FSGI score of 9 or less has been reported to have a 78% likelihood of survival [16].

The time to treatment is paramount for a chance at a favorable prognosis. Delays in the presentation of symptoms, delays in seeking care, clinical recognition, logistical challenges (such as scheduling or competing emergencies), and transfer time from tertiary or resource-challenged facilities to institutions with resources are all factors that lower survival. Ideally, both the debridement and limb revascularization surgeries should be performed at the same time. However, close access to the surgical sites and patient positioning on the operating table while maintaining sterility would be a difficult challenge to overcome. If limb ischemia is deemed irreversible, then sepsis/gangrene should be treated first. If the clinical picture suggests that the limb can be salvaged, then vascular intervention can take precedence with shared decision-making.

Similar to this case, a 57-year-old German patient presented with bilateral ischemic legs and gangrene of both legs [17]. For their patient, a palliative course was charted. In our decision-making, limb salvage was considered due to the patient’s Rutherford IIb classification. Vascular surgery deemed that there was a reasonable chance of limb salvage and proceeded with revascularization first. The extensive proximal and distal occlusions in the femoral profunda femoris artery bilaterally with heterogeneous areas of calcification suggested a thrombotic-embolic etiology of the occlusion in the left leg. This, coupled with the prolonged ALI (i.e., greater than six hours), resulted in unsuccessful revascularization. Despite interim clinical improvement, our attempts at revascularization and salvaging the limb and multiple debridements of the perineal and deep presacral tissues were unsuccessful, as the patient died. In retrospect, limb amputation would be warranted and could have been done to prevent repeated trips to the operating table, as well as decrease total operation time. A palliative/hospice course could have been presented earlier to the family as an alternative; however, the patient’s initial presentation and lack of significant comorbidities made it reasonable to attempt surgical salvage.

## Conclusions

Our patient originally presented with signs and symptoms of acute on chronic limb ischemia and experienced a delay in care as his acute limb ischemia was later discovered to be due to his fulminant Fournier’s gangrene. Due to the extensiveness of his disease and despite the intraoperative care and interim improvements, the patient lost his limb and ultimately, his life. In the context of an aging US population, those with known risk factors and comorbidities such as hypertension, atherosclerotic disease, poor social determinants of health, a history of smoking, sedentary work and/or lifestyle, and patients with a history of delay in seeking care should be evaluated with a higher index of suspicion for underlying life-threatening causes. Here, early recognition becomes the key determinant of limb revascularization and rescue. For patients such as in the case presented here, i.e., a high-risk profile for comorbid conditions, regardless of which issue is addressed first, a multidisciplinary team of general surgeons, vascular surgeons, and intensivists should be applied in order to create a treatment plan that addresses the critical issues in the most appropriate order.
